# Single-cell transcriptomes and T cell receptors of vaccine-expanded apolipoprotein B-specific T cells

**DOI:** 10.3389/fcvm.2022.1076808

**Published:** 2023-01-05

**Authors:** Felix Sebastian Nettersheim, Yanal Ghosheh, Holger Winkels, Kouji Kobiyama, Christopher Durant, Sujit Silas Armstrong, Simon Brunel, Payel Roy, Thamotharampillai Dileepan, Marc K. Jenkins, Dirk M. Zajonc, Klaus Ley

**Affiliations:** ^1^La Jolla Institute for Immunology, La Jolla, CA, United States; ^2^Department of Cardiology, Faculty of Medicine and University Hospital Cologne, University of Cologne, Cologne, Germany; ^3^Department of Microbiology and Immunology, University of Minnesota Medical School, Minneapolis, MN, United States; ^4^Department of Bioengineering, University of California, San Diego, San Diego, CA, United States; ^5^Immunology Center of Georgia (IMMCG), Augusta University, Augusta, GA, United States

**Keywords:** ApoB, P6, atherosclerosis vaccine, regulatory T cells, single-cell RNA-sequencing

## Abstract

Atherosclerotic cardiovascular diseases are the major cause of death worldwide. CD4 T cells responding to Apolipoprotein B (ApoB), the core protein of most lipoproteins, have been identified as critical disease modulators. In healthy individuals, ApoB-reactive (ApoB^+^) CD4 T cells are mostly regulatory T cells (T_regs_), which exert anti-inflammatory effects. Yet, they may obtain pro-inflammatory features and thus become proatherogenic. Evidence from animal studies suggests that vaccination against certain major histocompatibility complex (MHC) II-binding ApoB peptides induces an expansion of ApoB^+^ T_regs_ and thus confers atheroprotection. To date, in-depth phenotyping of vaccine-expanded ApoB^+^ T cells has not yet been performed. To this end, we vaccinated C57BL/6J mice with the ApoB-peptide P6 (ApoB_978–993_ TGAYSNASSTESASY) and performed single-cell RNA sequencing of tetramer-sorted P6^+^ T cells. P6^+^ cells were clonally expanded (one major, two minor clones) and formed a transcriptional cluster distinct from clusters mainly containing non-expanded P6^+^ and P6^–^ cells. Transcriptomic profiling revealed that most expanded P6^+^ cells had a strong T_reg_ signature and highly expressed genes mediating suppressive functions. Yet, some expanded P6^+^ cells only had a residual T_reg_ signature and expressed genes related to T helper 1 (T_H_1) cells, which are proatherogenic. Modeling the T cell receptor (TCR) and P6:MHC-II interaction showed that only three amino acid residues in the α and β chain contact the P6 peptide in the MHC-II groove and thus determine the specificity of this TCR to P6. Our data begin to reveal the vaccination-induced response to an ApoB epitope.

## 1. Introduction

Atherosclerosis, the major underlying cause of cardiovascular diseases, involves chronic inflammatory processes in the arterial wall which are mediated by an autoimmune response against plaque-associated autoantigens (1, 2). Accumulating evidence suggests a critical role of CD4 T cells responding to Apolipoprotein B (ApoB), the core protein of most lipoproteins, in atherogenesis (3). ApoB reactive (ApoB^+^) CD4 T cells have mixed phenotypes in healthy mice and humans that undergo dynamic changes in the course of atherosclerosis development (4, 5). Initially, ApoB^+^ T cells with regulatory T cell (T_reg_) signatures dominate. Regulatory T cells (T_*regs*_) are one of the lineages of CD4 T cells that limit inflammatory responses (6) and thereby confer atheroprotection (7, 8). During atherogenesis, ApoB^+^ T cells progressively lose their T_reg_ signatures and acquire phenotypes with pro-inflammatory properties (4, 5).

Preclinical studies have shown that vaccination against certain ApoB peptides induces an expansion of T_*regs*_ and thereby protects from atherosclerosis (9). Most of these studies were limited as epitope-specific CD4 T cells were not identified and, consequently, the underlying mechanisms of ApoB-vaccine-related tolerogenic immunity are not well understood. CD4 T cells recognize peptide epitopes bound to major histocompatibility complex class II (MHC-II) (10). Mouse models on the C57BL/6J genetic background express only one single allele of MHC-II, I-A^b^, which enables detection of antigen-specific CD4 T cells by using recombinant truncated I-A^b^ molecules covalently loaded with the antigenic peptide and tetramerized by fluorescently labeled streptavidin (11). We recently detected T cells reactive to the ApoB epitopes P6 and P18 by using I-A^b^ tetramers (4, 12). In line with previous analyses of bulk CD4 T cells (13–15), flow cytometry suggested that vaccination against ApoB peptides mainly induces an expansion of ApoB^+^ T_*regs*_ (12). To date, in-depth characterization of vaccine-expanded ApoB^+^ T cells has not been performed.

Most CD4 T cells express only one αβ heterodimeric TCR per cell. Both TCRα and TCRβ are highly polymorphic, with billions of sequences generated by V(D)J recombination and template-free filling to generate the CDR3 sequences, which are largely responsible for antigen specificity. Single cell RNA sequencing (scRNA-Seq) can be used to assemble paired TCRα and β chains (16–18). Single cell TCR sequences of ApoB-specific CD4 T cells have not been described.

Here, we combined P6: I-A^b^ tetramer sorting with Smart-Seq2 scRNA-Seq (single-cell single-well) (19) to obtain full-length transcriptomes and paired TCRα and β chains of P6^+^ CD4 T cells. This method is more expensive and produces fewer (tens to hundreds) but much better transcriptomes than alternative methods (20, 21). Our data show that immunization with P6 induces oligoclonal expansion of P6^+^ T cells with a dominating T_reg_ signature. We identified upregulation of several genes involved in mediating suppressive functions. Our findings are the first full-length transcriptomes and paired α/β TCR sequences from *ex vivo* T cells specific for an atherosclerosis-related antigen and will help to better understand the autoimmune response in atherosclerosis.

## 2. Materials and methods

### 2.1. Animals and experimental design

Seven-week-old C57BL/6J female mice were purchased from The Jackson Laboratory (strain# 000664 Bar Harbor, ME, USA) and maintained under specific pathogen-free conditions. All animal studies were approved by the local Institutional Animal Care and Use Committee. ApoB_978–993_ (P6: TGAYSNASSTESASY) peptide was purchased from A&A Labs (San Diego, CA). Complete (CFA) and incomplete (IFA) Freund’s adjuvants were purchased from SIGMA (St. Louis, MO). C57BL/6J mice were intramuscularly immunized with 100 μg P6 peptide in 100 μl CFA/PBS (50:50%) at week 0 and 100 μg P6 peptide in 100 μl IFA/PBS (50:50%) at week 2. For each immunization 50 μl were injected into the left and right musculus quadriceps femoris. Two weeks after final immunization the inguinal and para-aortic lymph nodes were collected.

### 2.2. P6 tetramer production

ApoB:MHC monomers were expressed as previously described (11). Briefly, sequences encoding the antigenic peptide ApoB 978–993 were fused to the N-terminus of the mouse MHC-II (I-A^b^) beta chain by a flexible polyglycine linker in the pRMHa-3 expression vector and co-expressed in Drosophila melanogaster S2 cells with the mouse MHC-II (I-A^b^) alpha chain and BirA ligase. Biotinylated ApoB:MHC monomers were purified from culture supernatants using nickel affinity chromatography, followed by an additional purification on a Pierce Monomeric Avidin UltraLink Resin column (Thermo Fisher Scientific, Waltham, MA, USA) and coupled to streptavidin-phycoerythrin (PE) or streptavidin-allophycocyanin (APC) (Prozyme, Hayward, CA, USA) to generate tetramers.

### 2.3. Peptide:MHC tetramer staining and index sorting

Cell suspensions were prepared from draining (inguinal and para-aortic) lymph nodes. Lymph nodes were passed through a 70 μm cell strainer and the cell suspension was filtered once more through a 70 μm strainer before washing (400 × g, 4 min, room temperature). Cells were counted using trypan blue and a Neubauer chamber. Before incubation with tetramers, CD4 + T cells were enriched by a negative magnetic bead separation followed by anti-mouse biotinylated monoclonal antibodies all obtained from Tonbo Biosciences, San Diego, CA, USA:

CD8α, clone 53-6.7, cat.# 30-0081-U500; CD19 clone 1D3, cat.# 30-0193-U500; NK1.1, clone PK136, cat.# 30-5941-U500; CD11c, clone N418, cat.# 30-0114-U500; F4/80, clone BM8.1, cat.# 30-4801-U500; CD11b clone M1/70, cat.# 30-0112-U500; TER-119, clone TER-119, cat.# 30-5921-U500; CD45R, clone RA3-6B2, cat.# 30-0425-M001; and streptavidin-coupled magnetic microbeads (Stem Cell Technologies, Seattle, WA, USA). 20 μl of each antibody and 70 μl magnetic beads were used for each mouse.

Enriched CD4 + T cells were > 90% pure, based on surface expression of TCR-β and CD4 as measured by flow cytometry. Isolated CD4 T cells were washed (400 × g, 4 min at 4°C) and incubated for 30 min at 37°C and 5% CO_2_ in 100 μl of 50 nM Dasatinib (Stem Cell Technologies) in RPMI supplemented with 10% FCS (Gemini Bio, West Sacramento, CA, USA) and 1x Pen/Strep (Thermo Fisher Scientific). Subsequently, 1 μl (equaling a final concentration of 10 nM) of ApoB:MHC-streptavidin-PE and ApoB:MHC-streptavidin-APC tetramer were added for an additional 45 min incubation period. Cells were washed (400 × g, 4 min at 4°C), the supernatant discarded and resuspended in 100 μl of Live/Dead Aqua (Thermo Scientific Fisher) diluted 1:1,000 in PBS. After a 30 min incubation on ice in the dark, 100 μl of staining buffer (fetal bovine serum 1:50 diluted in PBS) were added to the samples and cells were washed (400 × g, 4 min at 4°C). The supernatant was discarded and the cells were resuspended in staining buffer with the following anti-mouse monoclonal antibodies at 1:100 dilution:

CD16/32, clone 2.4G2, Tonbo Biosciences, cat.# 70-0161-M001; CD8a BV421, clone 53-6.7, BioLegend, San Diego, CA, USA, cat.# 100738; CD19 BV421, clone 6D5, BioLegend, cat.# 115538; NK1.1 BV421, clone PK136, BioLegend, cat.# 108732; CD11c BV421, clone N418, BioLegend, cat.# 117343; F4/80 BV421, clone BM8, BioLegend, cat.# 123132; CD11b BV421, clone M1/70, BioLegend, cat.# 101251; Ter119 BV421, clone TER-119, BioLegend, cat.# 116234, CD45R BV421, clone RA3-6B2, BioLegend, cat.# 103251; CD25 BV605, clone PC61, BioLegend, cat.# 102036; CD62L BV785, clone MEL-14, BioLegend, cat.# 104440; TCR-β Alexa Fluor 700, clone H57-597, BioLegend, cat.# 109224; CD4 APC-eFluor 780, clone GK1.5, Thermo Fisher Scientific, cat.#47-0041-82; CD44 PE/Cy7, clone IM7, BioLegend, cat.# 103030.

After staining for 30 min on ice in the dark, cells were washed (400 × g, 4 min at 4°C), the supernatant was discarded, and cells were resuspended in 150 μl staining buffer for sorting with a BD Aria Fusion (BD Biosciences, San Diego, CA, USA). A 70 μm nozzle and medium pressure at 800–1,200 events/s were used to index sort single cells into single wells of a 384-well plate (Thermo Fisher Scientific, 4483322, MicroAmp EnduraPlate optical). For sorting P6:MHC-positive CD4 T cells, 90% of the sample volume were used while P6:MHC-negative CD4 T cells were sorted from the remaining 10%. Each well contained 4 μl lysis buffer. For 1 reaction of lysis buffer 2 μl of diluted RNase inhibitor, 1 μl dNTPs (10 mM, Thermo Fisher Scientific) and 1 μl of 10 μM Oligo-dT30VN (IdT, Coralville, IA, USA) were mixed. RNase inhibitor (Takara/Clonetech, Mountain View, CA, USA) was diluted 1:20 in 0.2% Triton X-100 (Merck, Darmstadt, Germany), which was diluted in nuclease-free water (Qiagen, Hilden, Germany)

After sorting, the plate was sealed, gently vortexed, and centrifuged at 2,000 rpm for 30 s ensuring that all cells were collected in the lysis buffer. Subsequently, the plate was incubated for 3 min at 72°C to hybridize the Oligo-dT primer with the mRNA. The plate was immediately stored at -80°C until library preparation.

### 2.4. Library preparation

Single cell libraries were prepared according to the Smart-Seq2-protocol (22, 23) with the following modifications. Pre-amplification PCR cycles were increased to 23 to obtain sufficient amounts of cDNA for the sequencing analysis. Primer dimers were eliminated by two 0.8x Ampure-XP bead clean ups. 0.3–0.5 ng of pre-amplified cDNA were subjected to library preparation with the Nextera XT library preparation kit, Illumina, San Diego, CA, USA in 8 μl reaction volume. Barcoded libraries were pooled and sequenced the with a S1 flow cell and a 300-cycle kit on a NovaSeq Illumina platform to obtain 150-bp paired-end reads. Quality controls were performed using TapeStation with D5000 high sensitivity tapes (both from Agilent, Santa Clara, CA, USA). Quantification was performed using Qubit high sensitivity kit (Thermo Fisher Scientific) after PCR preamplification, and the PicoGreen assay (Thermo Fisher Scientific) after Nextera XT had been performed.

### 2.5. Preprocessing and quality control

A total of 150 bp paired-end raw sequencing reads from Illumina NovaSeq (Illumina pipeline v1.9) for 176 cells were run through FASTQC^1^ (v0.11.7). All cells passed sequence quality score as defined by FASTQC. On average, we captured 2.5 million reads per cell. STAR (v2.6.0a) (24) index was built using ENSEMBL GRCm38 genome (primary assembly) and annotation (GRCm38.92) (25). Raw reads were then mapped using STAR with the added parameters “–quantMode GeneCounts” and “–outSAMunmapped Within”. The mapped reads BAM file was sorted using SAMtools (v0.1.19) (26). Post-mapping sequencing quality was performed on the sorted BAM file using QoRTs (v1.3.0) (27). Gene body coverage was determined for the upper-middle quartile genes according to QoRTs. Mapping rate includes unique as well as multi-mapping reads.

### 2.6. scRNA-Seq analysis

Gene counts were obtained from STAR. ENSEMBL gene ids were converted to gene names using the ENSEMBL annotation (25). Genes which are not within chromosomes were discarded. Ensembl gene ids without a corresponding gene name were discarded. Identical gene names were collapsed using average. Seurat (28) was used to analyze the scRNA-Seq dataset. We discarded cells with less than 500,000 reads, or less than 2,000 genes, cells with high mitochondrial content (= 10%), or cells with low total mapping rate (=75%). All analyses were performed using the Seurat (v3.0.2) R package. The expression of the remaining 129 cells were normalized (LogNormalization with scale.factor = 1e6) and imputed using ALRA. The top 500 variable genes using the “vst” method were selected and scaled, regressing out the effects of mitochondrial content, plate, and sequencing depth. PCA was performed and the first 8 PCs according to JackStraw method were retained. UMAP (29) projection was done on these PCs, with number of neighbors set to 50. Clustering was done using Louvain algorithm, with resolution set to 0.3 and k.param for neighbor finding set to 10. Marker identification was done using the ROC test, retaining only up-regulated genes with an AUC = 0.8. g:Profiler (30) was used to find over-represented GO terms and pathways in the marker genes. Statistically significant pathways with a q-value of =0.1 are reported. Raw FASTQ files were fed into TraCeR (v0.6.0) (16) “assemble” to reconstruct the alpha and beta chains of the TCR. Default arguments were used. We used all the reconstructed chains to call TCR clonotypes regardless of productivity or expression level. Heatmaps were created using pheatmap (v1.0.12). Chain similarity graph was done using ggraph (v1.0.2).

### 2.7. Structural modeling

The three I-A^b^ crystal structures PDB ID 3C60, 2IAD, and 1AIO were superimposed, revealing conserved backbone binding of the three different peptides in the I-A^b^ binding groove. We used NetMHCIIpan (v3.2) (31) to predict the binding core of the ApoB6 peptide, which was then superimposed with the binding cores of the three peptides from the available crystal structures. The ApoB6 peptide was manually built in COOT (32) to identify possible TCR contact residues.

We next superimposed five complex crystal structures with different I-A restricted TCR’s which demonstrated a common mode of binding above the I-A^b^ peptide binding groove. Of all TCRs, the β chain was more conserved in sequence compared to the α chain. The TCRs bound to I-A^b^ with similar footprint. Thus, we assumed that the β chain dictated the conserved binding orientation and, therefore, we modeled our β chain on top of the TCRβ chain that has the highest sequence identity. A multiple sequence alignment of these TCRs with the major clone (TRAV7D-6/TRAJ17, TRBV13-2, TRBJ2-4) identified in this study revealed an almost conserved sequence with the TCRβ chain of YAe62 (33) (PDB ID 3C60), with the exception of CDR3β. We modeled our TCR sequence by superimposition with the TCRβ chain of YAe62 and manually rebuilt the CDRs, where they differed in length and sequence, to identify possible contact residues with the ApoB6 peptide. The B3K506 (PDB ID 3C5Z) TCRβ chain was slightly more diverse in sequence and was excluded as a template for modeling.

## 3. Results

### 3.1. Single-cell sequencing and TCR reconstruction reveals clonal expansion of vaccine-expanded ApoB^+^ CD4 T cells

**FIGURE 1:**
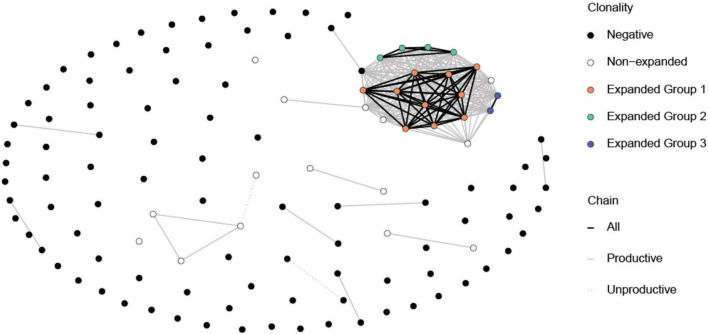
Common TCR α and β chains among the P6^+^ and P6^–^ cells. Each circle represents a cell. The expanded P6^+^ cells (orange, green and blue circle) have at least one chain in common amongst themselves. A few non-expanded P6^+^ cells also have some common chains with expanded P6^+^ cells even though they may not belong to the same clonotype. Expectedly, P6^–^ cells mostly do not have chains in common. Lines represent at least one common chain between the cells at its termini. Line color represents which chain (s) is shared. Clonotypes were based on having the exact same reconstructed chains for both productive and non-productive chains. Cells with no successful reconstructed chains were not included in this figure.

**FIGURE 2:**
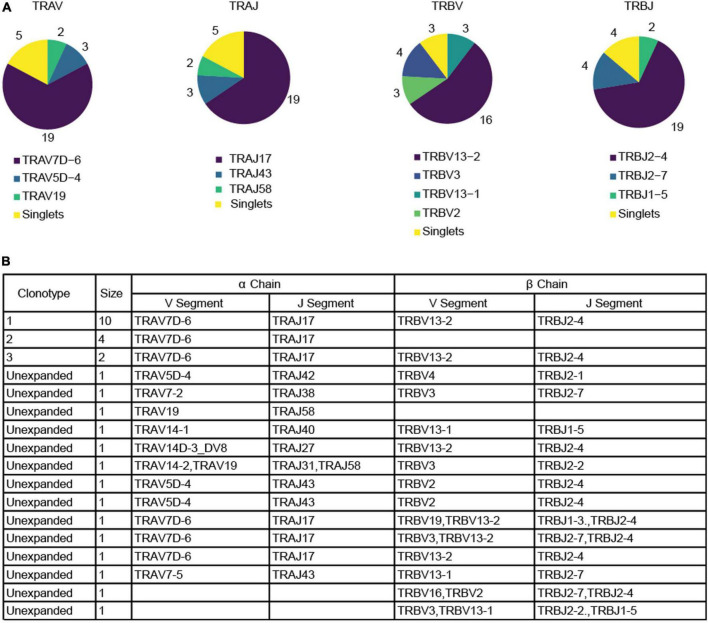
Productive α and β chain reconstruction for the P6^+^ cells. **(A)** Pie charts depicting the frequency of each gene for V and J segment in both productive chains among tetramer^+^ cells. **(B)** Table listing the reconstructed genes for each productive chain among P6^+^ cells as well as the size of the clonotype. Blank fields represent cases in which the reconstruction algorithm failed to reconstruct a chain. Two unexpanded cells were excluded because no productive chains were identified. Clonotype calling was based on both productive and non-productive chains.

We immunized C57BL/6J mice with ApoB P6 (ApoB978-993 TGAYSNASSTESASY) in CFA i.m. followed by P6 in IFA 2 weeks later and harvested the draining (inguinal and para-aortic) lymph nodes at 4 weeks after the primary immunization (Supplementary Figures 1A–C). Antigen-experienced CD4 T cells were identified as TCRβ^+^CD4^+^CD44*^hi^*CD62L^–^. All 32 P6:I-A^b^ tetramer-PE and -APC positive cells (P6^+^) from one ApoB-P6 immunized mouse and 144 tetramer-negative antigen-experienced CD4 T cells were sorted into single wells. Next, libraries were prepared (NexteraXT) and sequenced. All P6^+^ and 97 out of 144 P6^–^ cells passed quality filters (Supplementary Figures 2, 3). TraCeR was used for TCR assembly (16). In 23 out of 32 P6^+^ cells (71%) and in 53 out of 97 P6^–^ cells (54%), both TCRα and β chains were successfully reconstructed. Both productive (expressed) and non-productive rearrangements were used to call clonotypes (Figure 1 and Supplementary Table 1). Clonal expansion (one major, two minor clones) was identified in the P6^+^ cells. Although considered a different clonotype (based on productive and non-productive α and β chains), several other unexpanded cells shared the same α or β chain with that major clone (Figure 1). The most common ApoB-P6-specific TCRα and β chains contained TRAV7D-6 and TRAJ17 as well as TRBV13-2 and TRBJ2-4 (Figure 2 and Supplementary Table 1).

### 3.2. Expanded ApoB^+^ T cells form transcriptional clusters and upregulate T_reg_ marker genes

**FIGURE 3:**
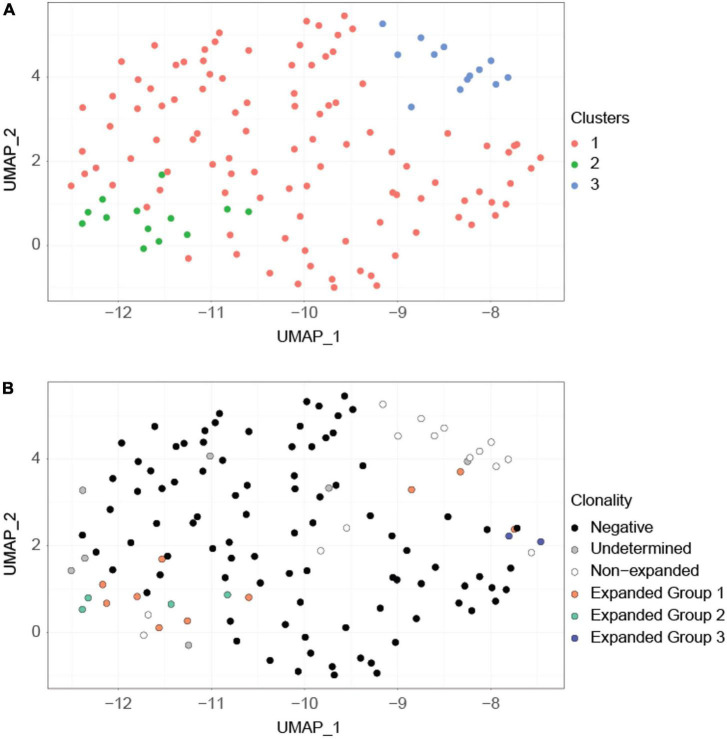
Clustering based on transcriptional profiles correlates with clonality. **(A)** UMAP projection of the cells. This projection is based on the first 8 principal components of the normalized imputed expression data of the highly variable genes. Graph-based clustering revealed three major clusters (orange, green, blue). **(B)** The same UMAP projection but with cells labeled according to their clonotypes. Expanded P6^+^ cells are separated into three clonotypes (orange, green, blue) based on their productive and non-productive TCRα and TCRβ chains. Expanded group 3 (blue) cells are separated from the remainder of the expanded P6^+^ cells. Undetermined cells are cells for which TCR information is unavailable.

Clustering of cells according to their transcriptional profile revealed three clusters (Figure 3A). Although these transcriptional clusters were not clearly separated, overlaying the TCR clonotype information revealed good matching to groups composed mostly of expanded P6^+^ cells, non-expanded P6^+^ cells and P6^–^ cells (Figure 3B). Unlike the major expanded clone (10 cells; orange) and the moderately expanded clone (4 cells; green) which were in the expanded P6^+^ cluster, the minor expanded clone (2 cells; blue) was among the P6^–^ cluster (Figure 3B). Expression of *Foxp3*, the lineage-defining transcription factor (TF) of T_*regs*_, and several other T_reg_-related genes such as the co-inhibitory molecule cytotoxic T-lymphocyte associated protein 4 (*Ctla4*), the TF helios (*Ikzf2*), the tumor necrosis factor receptor superfamily member 18 (*Tnfrsf18*, CD379), or the capping actin protein (*Capg*) were upregulated in expanded compared to non-expanded P6^+^ cells (Supplementary Table 2). Gene Ontology (GO) enrichment analysis further revealed that the expanded P6^+^ cell marker genes were enriched for terms related to lymphocyte development, T cell activation, cellular response, transport and fiber organization (Supplementary Table 3).

### 3.3. Hierarchical clustering confirms a T_reg_ phenotype of expanded ApoB^+^ T cells

**FIGURE 4:**
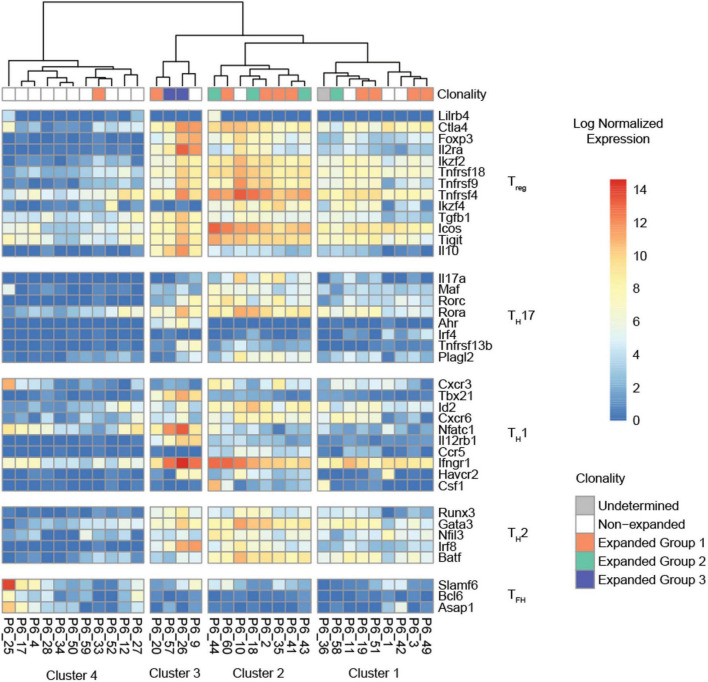
Hierarchical clustering of the normalized expression heatmap of 38 lineage-defining genes across all P6^+^ cells. Columns were clustered according to Ward’s second clustering criterion. Four segments were automatically generated based on the hierarchical clustering. All clusters with expanded P6^+^ cells (clusters 1, 2, 3) show evidence of a T_reg_ signature.

We compiled a list of 39 T_reg_, T helper 1 (T_H_1), 2 (T_H_2), 17 (T_H_17), and T follicular helper cell (T_FH_) lineage-defining genes that were highly expressed [imputed counts per million (cpm) =10 in at least three cells] to determine the lineage of each individual P6^+^ cell. Hierarchical clustering based on these genes showed four clearly distinguishable transcriptomic profiles (Figure 4). Most of the expanded P6^+^ cell transcriptomes were in clusters 1 and 2. Cells in cluster 2 highly expressed 11 of 12 T_reg_ genes including *Foxp3*, *Ctla4*, *Ikzf2*, *Tnfrsf18*, and *Il2ra*, the gene encoding the high affinity IL-2 receptor CD25. Additionally, a few T_H_17 genes were expressed by the cells in cluster 2, most prominently *Rora* and, to a lesser extent, *Rorc*, which encodes the lineage-defining TF RORγT. The cells in cluster 2 also expressed some T_H_2 genes, such as *Gata3*, which encodes the lineage-defining TF GATA3, and the TFs *Batf* and *Runx3*. In summary, this gene signature defines cluster 2 as expanded and effector T_regs_ (Figure 4). The transcriptomic profile of cluster 1 was similar to cluster 2, although the T_reg_ signature was weaker. Four P6^+^ cells formed cluster 3. These cells had a clear residual T_reg_ signature, but highly expressed T_H_1-related genes such as the *Tbx21*, encoding the lineage-defining TF T-bet, interferon gamma receptor 1 (*Ifngr1*), or nuclear factor of activated T-cells, cytoplasmic 1 (*Nfatc1*). In addition, this cluster showed particularly high expression of T_reg_ marker genes that are also markers of T cell activation (*Ctla4*, *Il2ra*) (34) as well as genes encoding the T_reg_-related cytokines transforming growth factor beta 1 (*Tgfb1*) (35) and interleukin 10 (*Il10*) (36). The T_H_17 TF *Rora* was still expressed, whereas the T_H_2 signature was weaker compared to cluster 2. Cluster 4 was largely populated by unexpanded P6^+^ cells and contained only one cell from expanded group 1 (orange). These cells showed a weak residual T_reg_ signature and low levels of *Rora* as well as *Ifngr1*. The T_H_2 signature was largely lost. Some of these cells expressed the T_FH_ marker genes *Slamf6* and *Asap1*.

### 3.4. The T-cell receptor specificity to P6 is determined by three amino acid residues

**FIGURE 5:**
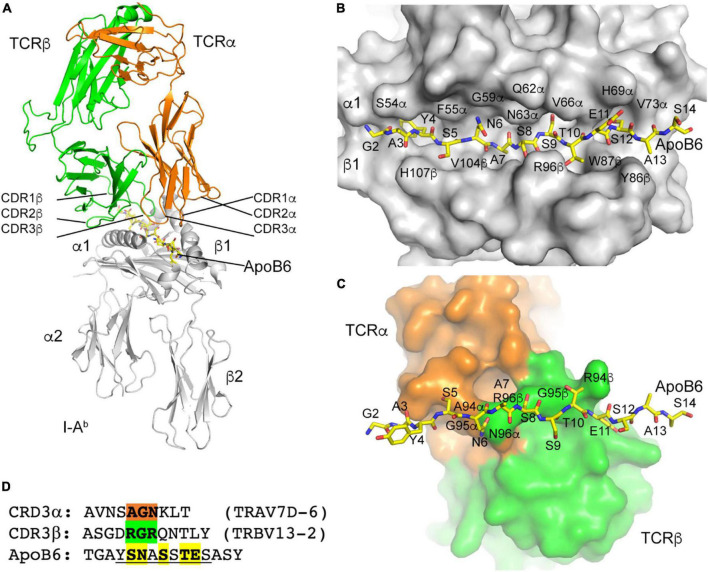
I-A^b^ ApoB P6 peptide and TCR modeling. **(A)** Overview of the TCR/I-A^b^/ApoB P6 complex with I-A^b^ in gray, TCRα in orange and TCRβ in green. ApoB P6 peptide in yellow sticks. **(B)** Binding of the ApoB P6 peptide in the I-A^b^ binding groove identifies amino acid residues that are accessible for TCR binding. **(C)** TCR-ApoB P6 contact residues. **(D)** TCRα contact residues highlighted orange and TCRβ residues green. The ApoB P6 core peptide is underlined and contact residues are highlighted yellow.

Having successfully reconstructed the α and β TCRs of 32 P6^+^ CD4 T cells, we threaded the most abundant clone (Vα7D-6Jα17 Vβ13-2Jβ2-4, 10 cells) through the published crystal structure of mouse TCR YAe62 (pdb 3C60). CDR1, 2, and 3 for both α (orange) and β (green) are shown in Figure 5A. The ApoB-P6 peptide was placed in the groove of the published crystal structures of mouse I-A^b^ (pdb 1AIO, 2IAD, and 3C60). As expected, residues Y4, A7, S9, and S12 in the 15-mer were anchor residues (Figure 5B), with the residues S5, N6, S8, T10, and E11 pointing “up” toward the TCR heterodimer (Figure 5B). Residues S5 and N6 contacted TCRα A94 and G95 (Figure 5C). S8 made contact with TCRβ G95. T10 made contact with TCRβ R94 (Figure 5C). Thus, three contiguous amino acid residues in CDR3α (AGN) and in CDR3β (RGR) determined the specificity of this TCR for P6 in the groove of I-A^b^ (Figure 5D).

## 4. Discussion

We and others have shown that a CD4 T cell response against MHC-II-restricted ApoB-peptides is critically involved in atherogenesis (4, 37, 38). Accumulating evidence suggests that immunization against such peptides leads to an expansion of ApoB-reactive T_*regs*_ and thereby affords considerable atheroprotection in mice (9, 39). Until now, vaccine-expanded ApoB^+^ T cells have been only phenotyped by flow cytometry and functional assays (12, 40). Given the low cell number, single-cell analyses of antigen-specific CD4 T cells are extremely challenging. Herein, we utilized Smart-Seq2 single-cell single-well RNA sequencing (22) to provide the first full-length transcriptomes (including paired TCR α and β chain sequences) of T cells expanded by immunization with the ApoB peptide P6. We detected P6^+^ CD4 T cells by utilization of a p6:MHC-II tetramer, which has been extensively validated in a previous study (4). Particularly, the tetramer colocalized with the TCR, the number of tetramer^+^ T cells was higher after immunization with p6 and adjuvants (CFA) vs. adjuvants alone, tetramer^+^ T cells were not detectable in BALBc mice that express a different MHC-II allele (I-A^e^ instead of I-A^b^ in C57BL/6J mice), tetramer binding correlated with a fluorescent marker of antigen-specific TCR signaling (Nur77-GFP) after vaccination with p6, and restimulation with p6 *in vitro* induced cytokine secretion (IL-17) in tetramer^+^ T cells (4).

In most cells, we were able to reconstruct TCRα and β chains and call clonotypes. TCR reconstruction confirmed clonal expansion of P6^+^ cells and identified TRAV7D-6 and TRAJ17 as well as TRBV13-2 TRBJ2-4 as the most common P6-specific variable and junction TCRα and β chains, respectively. This contrasts with a study in which CD4 T cell hybridomas were generated from human ApoB-transgenic mice vaccinated with human oxidized LDL. In that study, the predominant Vβ was found to be TRBV31 (41). Possible explanations for this difference include that (1) the TCRβ usage during hybridoma generation may have been skewed, (2) the responses studied here may be specific to one ApoB peptide and other peptides may elicit different responses, or (3) the T cell response to human ApoB may be different from that to mouse ApoB. Identifying the Vα and β sequences enriched in expanded P6^+^ cells potentially (if suitable antibodies will be generated) enables detection of these rare cells without the use of tetramers and thereby can help better define the autoimmune response against ApoB in future studies. Every T cell develops a unique TCR through random genetic recombination during maturation in the thymus and, in contrast to the transcriptome of a T cell, the antigen specificity of the TCR is subsequently unaffected by external stimuli (42, 43). Consequently, our data can particularly help to identify P6^+^ cells in the setting of atherosclerosis.

Expanded ApoB^+^ cells exhibited higher expression of several T_reg_-related genes than non-expanded ApoB^+^ cells. Hierarchical clustering based on expression of lineage-defining genes revealed that most expanded ApoB^+^ cells had very similar phenotypes and, accordingly, clustered together. The dominating transcriptomic profile was characterized by an upregulation of selected T_reg_ marker genes, including the lineage-defining TF *Foxp3* (44) and *Il2ra*, which encodes for the high affinity IL-2 receptor CD25 (45, 46). Further T_reg_-related genes upregulated by expanded P6^+^ cells were *Ctla4* (47), *Ikzf2* (48), *Tnfrsf18* (49), *Tnfrsf9* (50), *Tnfrsf4* (51, 52), *Ikzf4* (53), *Tgfb1* (35), *Icos* (54), and *Tigit* (55). Cytotoxic T-lymphocyte-associated protein 4 (Ctla4) is a co-inhibitory molecule that is constitutively expressed in T_regs_ and critical for maintenance of their suppressive function (47). *Ikzf2* encodes for the TF Helios, which is also involved in maintaining a stable and suppressive T_reg_ phenotype (48). Tumor necrosis factor receptor superfamily member 18 (*Tnfrsf18*), also known as Glucocorticoid-induced tumor necrosis factor receptor-related protein (GITR), is highly expressed in Tregs and context-dependently modulates their function (49). The costimulatory molecule tumor necrosis factor receptor superfamily member 9 (*Tnfrsf9*), also known as 4-1BB/CD137, is directly controlled by Foxp3 (50) and mediates the suppressive function of T_regs_ (56). Tnfrsf4/OX40 marks effector T_regs_ and non-lymphoid tissue T_regs_ (51, 52). *Ikzf4* encodes the zinc-finger TF EOS, which mediates Foxp3-dependent gene silencing in T_regs_ and is important for maintenance of their suppressive function (53). *Tgfb1*, the gene encoding TGF-β is another important mediator of the suppressive activity of T_regs_ that limits autoimmunity (35). *Icos* (CD278) is a co-stimulatory molecule which has been implicated in T_reg_ development and functionality. Its presence is necessary to maintain numbers of peripheral T_regs_ while absence reduces demethylation of the T_reg_-specific demethylated region (TDSR) at the Foxp3 promotor leading to T_reg_ instability (54). *Tigit* marks a subset of T_regs_ which is capable of selectively suppressing T_H_1 and T_H_17 responses (55). In summary, almost all (94%) expanded P6^+^ cells were characterized by high expression of genes known to mediate the suppressive capacity of T_regs_. These findings are consistent with the idea that T_regs_ critically contribute to the atheroprotective effect of ApoB-related vaccines. The identified candidate genes will help to monitor T_reg_-related protective immunity during atherogenesis and in response to immunization strategies.

Most of the expanded P6^+^ cells additionally expressed a few T_H_17 genes, such as *Rora* and *Rorc*, which encodes the lineage-defining TF RORγT (57). RORγT-expressing T_regs_ are thought to be a specialized T_reg_ lineage induced by the gut microbiome (58, 59). We recently identified Foxp3 and RORγT co-expressing P6^+^ cells in humans and mice with atherosclerosis, although the specific role of this cell type in atherogenesis has not been clarified yet (4, 12, 60). All expanded P6^+^ cells also expressed *Ifngr1*, the gene encoding the Interferon gamma (IFNγ) receptor 1, which is a prototypical T_H_1 marker (61). Engagement of IFNγ with its receptor expressed on T_regs_ might drive their instability (62). Li et al. (63) and Butcher et al. (64) observed an increase in T_H_1 cells with residual expression of Foxp3 in mice with atherosclerosis. A small fraction of P6^+^ cells (19%, cluster 3) additionally expressed *Tbx21*, which encodes T-bet, the lineage-defining TF of T_H_1 cells. Additionally, these cells showed high expression of genes encoding T_reg_-related cytokines (*Tgfb1* and *Il10*) (34) and T cell activation marker genes (*Ctla4* and *Il2ra*) (34). Self-antigen-specific activation can induce instability of T_regs_ under specific conditions such as inflammation (65). Hence, the cells included in cluster 3 could have been about to switch from T_regs_ to T_H_1 cells, or they may be T_H_1T_regs_, which are also known as CCR5 T-effector cells (63, 64). Most expanded P6^+^ cells expressed *Gata3*, which encodes the T_H_2-lineage-defining TF GATA3 (66), and the T_H_2-related TF *Batf* (67). GATA3 is important for T_reg_ maintenance and functionality under inflammatory conditions and highly expressed by T_regs_ at barrier sites in the skin and the gastrointestinal tract (68). *Batf* controls T_H_2 cells bidirectionally: Depending on the binding partner it augments or inhibits T_H_2 cell differentiation and immune responses (67). Taken together, the majority of expanded P6^+^ cells co-expressed some markers typical for other T_H_-lineages. Our data may help to guide future studies investigating the role of such co-expression in the context of immunization against ApoB as well as atherogenesis in general.

Unexpanded P6^+^ cells exhibited low expression of most lineage-defining genes and might likely represent naïve T cells that have either not encountered P6 or were not sufficiently activated. Some of these cells expressed a T_FH_ profile, led by *Slamf6* (69) and *Asap1* (70), which is in accordance with a recent report that aortic T_regs_ can convert into T_FH_ cells (71).

Reconstruction of the TCR α and β chain allowed us to model and analyze the interaction between IA^b^-bound P6 and the most abundant TCR clone (TRAV7D-6, TRBV13-2). This modeling revealed that only three contiguous amino acid residues determine the TCR specificity for P6 (AGN and RGR in the TCR α and β chain, respectively). Knowing which features determine the TCR specificity to P6 can be a valuable resource for predicting and evaluating potential cross-reactivities between ApoB and other antigens in future studies.

In summary, our study identifies oligoclonal expansion of CD4 T cells in response to vaccination with ApoB-P6. Most of the clonally expanded cells expressed a clear T_reg_ signature and, particularly, showed an upregulation of genes involved in mediating the suppressive function. The successful reconstruction of TCRα and β in most cells, combined with the known peptide epitope defined by sorting with P6:I-A^b^ tetramer, provides complete structural information on an ApoB-specific TCR with peptide-loaded MHC-II. Our data is a resource for future studies investigating vaccination strategies with ApoB to modulate proatherogenic autoimmunity.

## Data availability statement

The data presented in this study are deposited in the GEO repository, accession number GSE221281.

## Author contributions

KL designed, supervised the study, and provided funding. FN, HW, and YG wrote the manuscript and prepared the figures. FN, HW, KK, CD, and SB performed the experiments. YG, DZ, SA, and PR analyzed the data. TD and MJ contributed to the research materials. All authors contributed to the data research, critically discussed the content, and reviewed the manuscript before submission and have read and agreed to the published version of the manuscript.

## Footnotes

1
https://www.bioinformatics.babraham.ac.uk/projects/fastqc/
