# Supplementary material for: Single-cell transcriptomes and T cell receptors of vaccine-expanded apolipoprotein B-specific T cells
Source: Front Cardiovasc Med. 2023 Jan 5;9:1076808. doi: 10.3389/fcvm.2022.1076808 (PMC9849899; doi:10.3389/fcvm.2022.1076808)
Supplement: Supplementary file 1 [file Data_Sheet_1.PDF]

Supplementary Material

Supplementary Material

Supplementary Figures

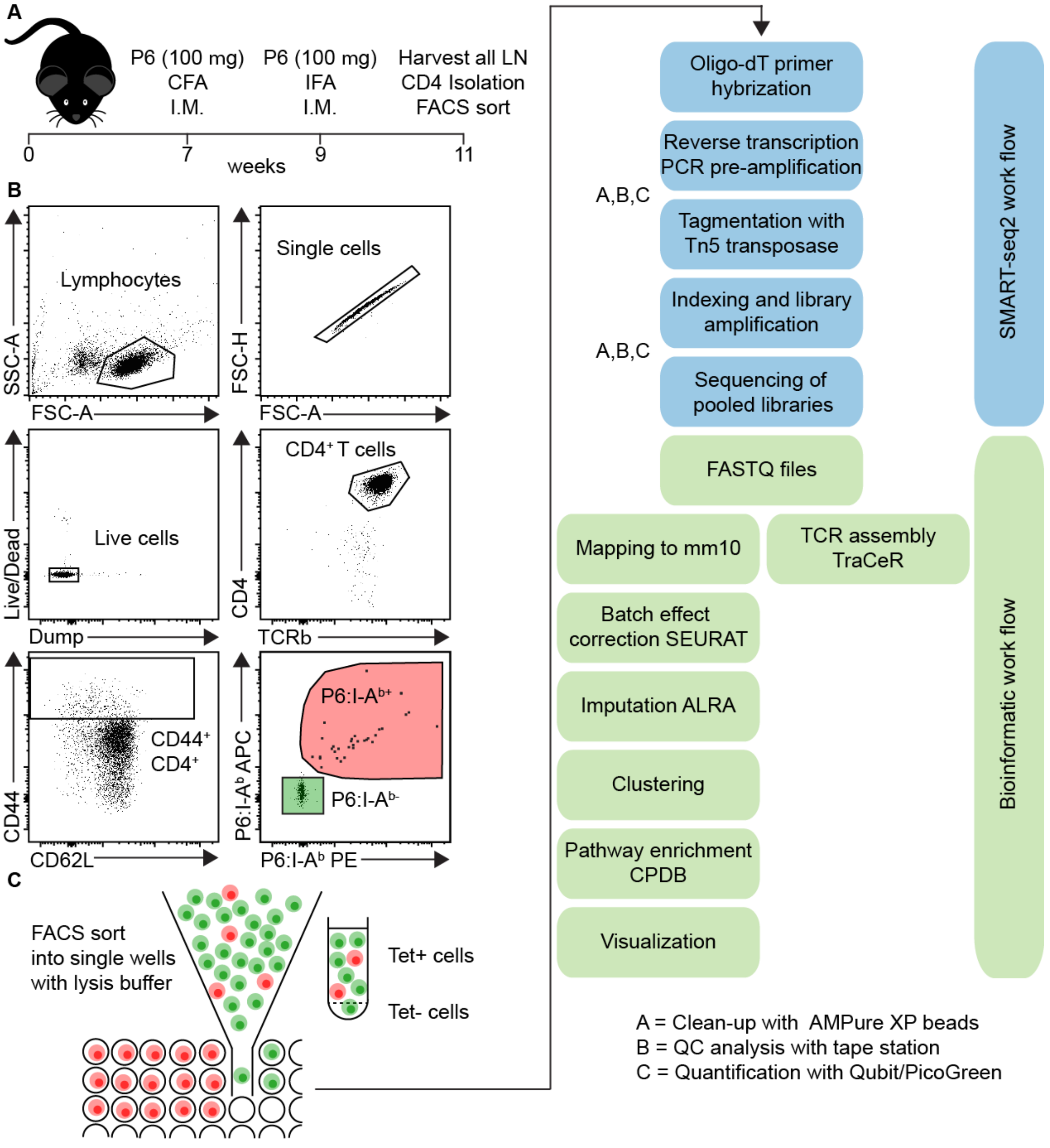

Supplementary Figure 1. Experimental design. (A) Female C57BL6/J mice were intramuscularly vaccinated with the Apolipoprotein B peptide P6 in complete and incomplete Freund's adjuvants (CFA

and IFA) at the age of 7 and 9 weeks, respectively. At 11 weeks of age, mice were sacrificed, CD4 T cells isolated from lymph nodes and incubated with two fluorochrome-conjugated MHC-II tetramers loaded with P6 peptide. P6:I-Ab^{+} and P6:I-Ab^{-} cells were isolated using the gating strategy for fluorescence activated cell sorting shown in (B). Single T cells were then subjected to a SMART-seq2 single transcriptional profiling protocol and bioinformatically analyzed according to the workflow depicted in (C).

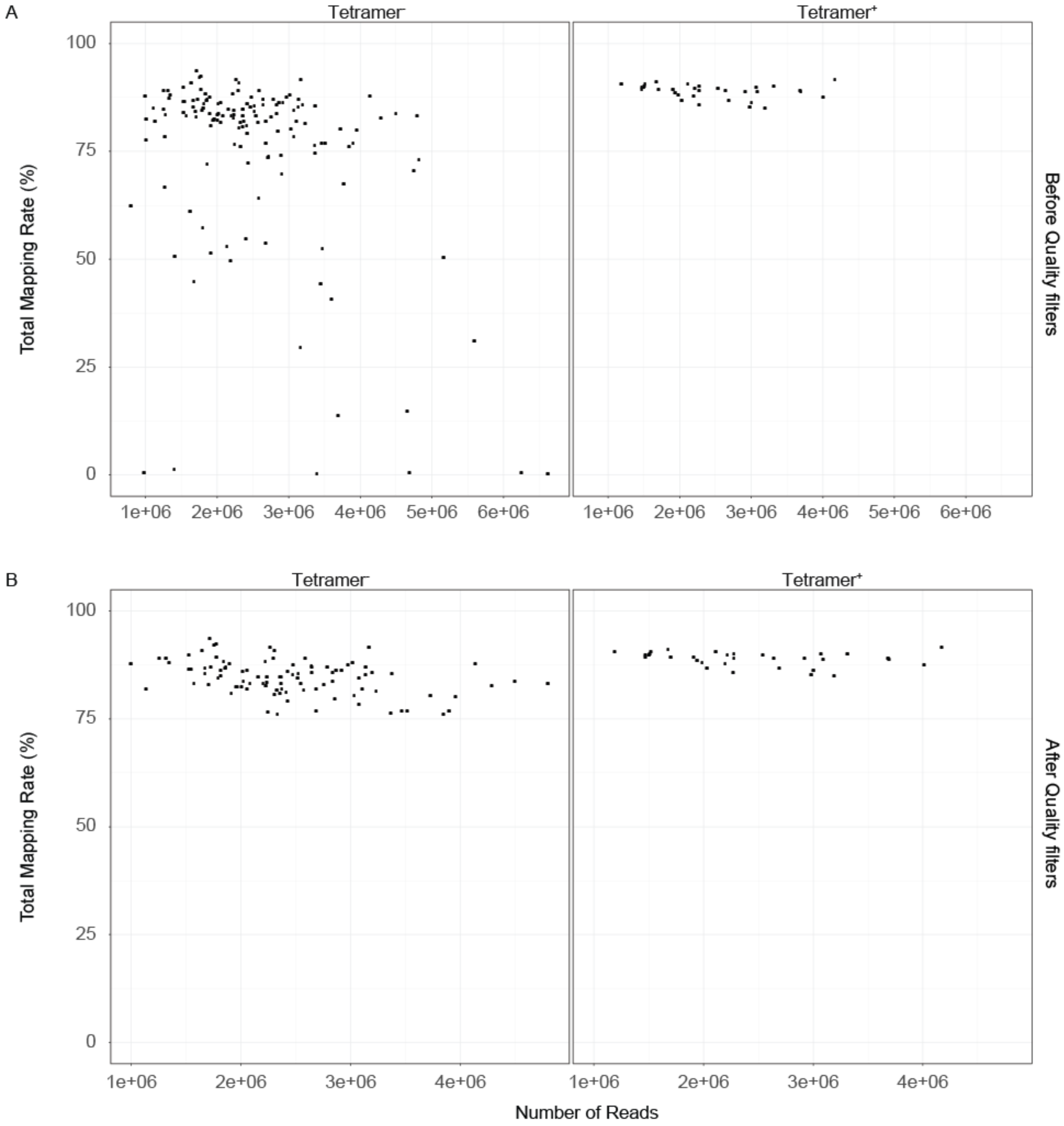

Supplementary Figure 2. Pre- and post- mapping quality controls on the cells. (A) Quality control for cells before filtering. Most Tetramer^{-} cells (left panel) and all Tetramer^{+} cells (right panel) had very good quality in terms of the total mapping rate and number of reads. (B) Quality control for

cells after filtering. All cells had at least 0.5 million reads and at least 75% total mapping rate. Total mapping rate refers to the sum of uniquely mapping and multi-mapping reads.

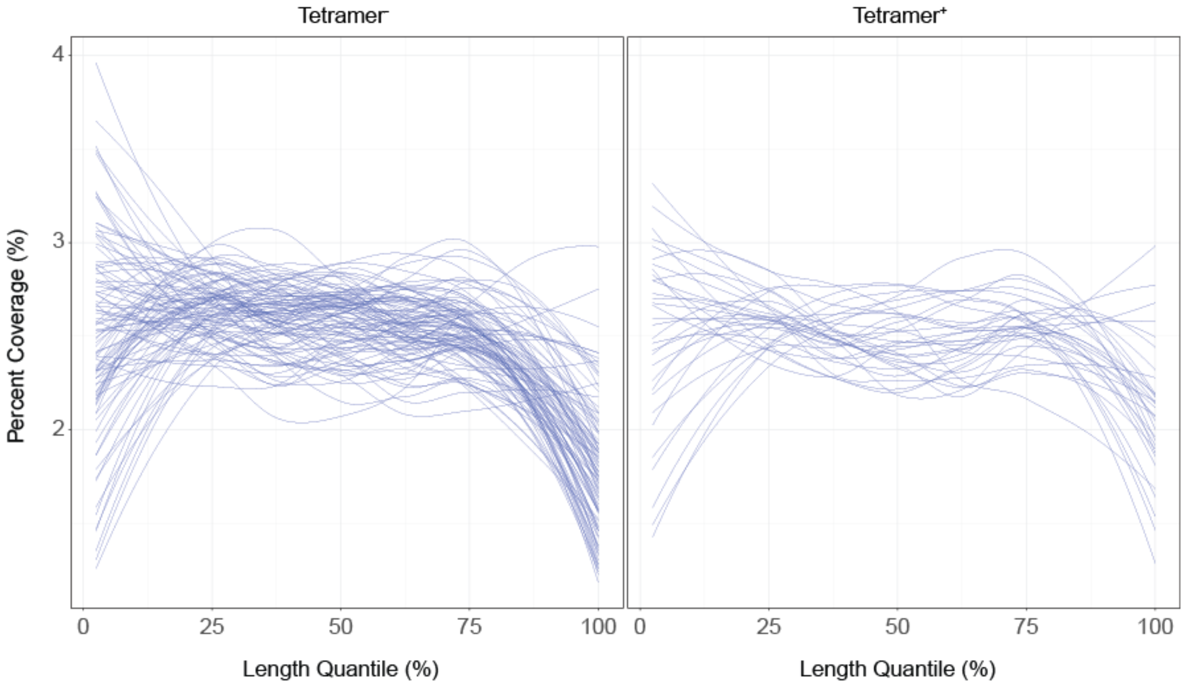

Supplementary Figure 3. Gene coverage for the Tetramer$^-$ and Tetramer$^+$ cells after filtering based on the output of QoRTs. Only the genes in the upper-middle quartile in terms of their expression were considered.
